# Relationship between lipid accumulation product and new-onset diabetes in the Japanese population: a retrospective cohort study

**DOI:** 10.3389/fendo.2023.1181941

**Published:** 2023-05-17

**Authors:** Ting Liu, Weilin Lu, Xiaofang Zhao, Tianci Yao, Bei Song, Haohui Fan, Guangyu Gao, Chengyun Liu

**Affiliations:** Department of Geriatrics, Union Hospital, Tongji Medical College, Huazhong University of Science and Technology, Wuhan, China

**Keywords:** lipid accumulation product, nonlinearity, diabetes, obesity, retrospective

## Abstract

**Background:**

Diabetes has become a global public health problem. Obesity has been established as a risk factor for diabetes. However, it remains unclear which of the obesity indicators (BMI, WC, WhtR, ABSI, BRI, LAP, VAI) is more appropriate for monitoring diabetes. Therefore, the objective of this investigation is to compare the strength of the association of these indicators and diabetes and reveal the relationship between LAP and diabetes.

**Methods:**

15,252 people took part in this research. LAP was quartered and COX proportional risk model was applied to explore the relationship between LAP and new-onset diabetes. Smooth curve fitting was employed to investigate the non-linear link between LAP and diabetes mellitus. Finally, the receiver operating characteristic (ROC) curve was used to evaluate the predictive ability of the aforementioned indicators for diabetes.

**Results:**

After adjusting for confounding factors, multiple linear regression analysis showed that each unit increase in LAP was associated with a 76.8% increase in the risk of developing diabetes (HR=1.768, 95% CI: 1.139 to 2.746, P=0.011). In addition, LAP predicted new-onset diabetes better than other indicators, and the AUC was the largest [HR: 0.713, 95% CI: 0.6806-0.7454, P<0.001, in women; HR: 0.7922, 95% CI: 0.7396-0.8447; P<0.001, in men]. When LAP was used as a lone predictor, its AUC area was largest both men and women. However, after adding classical predictors (FPG, HbA1c, SBP, exercise, age) to the model, the LAP is better than the ABSI, but not better than the other indicators when compared in pairs.

**Conclusions:**

High levels of LAP correlate very strongly with diabetes and are an important risk factor for diabetes, especially in women, those with fatty liver and current smokers. LAP was superior to other indicators when screening for diabetes susceptibility using a single indicator of obesity, both in men and in women. However, when obesity indicators were added to the model together with classical predictors, LAP did not show a significant advantage over other indicators, except ABSI.

## Introduction

With the rapid aging of the global population and the change in people’s lifestyles, diabetes has been recognized as one of the most prevalent diseases that endanger human health. By 2040, the number of people with diabetes is projected to explode to 642 million, according to the World Health Organization (1). Diabetes is a chronic disease that can lead to macrovascular and microvascular complications, including cardio-cerebrovascular disease (CVD), diabetic nephropathy (DKD), and diabetic retinopathy (DR). What’s more, diabetes can cause disability and reduce the quality of life (2). The early stage of diabetes lacks specific manifestations and patients have no obvious clinical symptoms, so diabetes is easy to be ignored in the early stage. Early detection of diabetes can promote early intervention, and early intervention can slow down or prevent the occurrence of complications, thus reducing the pain and medical burden of patients (3).

Of several controllable risk elements for type 2 diabetes, obesity has been identified as a major risk factor (4). Obesity indicators [Body Roundness Index (BRI), waist circumference(WC), lipid accumulation product (LAP), A Body Shape Index (ABSI), waist to height ratio (WHtR), body mass index (BMI), visceral Adiposity Index (VAI)] have been used to predict diabetes or metabolic syndrome in recent years. However, the ability of these obesity indicators to identify diabetes in clinical practice has been controversial in different trials. Several studies have shown that WC can be a preferred predictor of diabetes and metabolic syndrome compared to WHtR, BMI, BRI, ABSI, and LAP (5, 6). A study conducted on 3001 people in Ningbo, China, showed that BMI as an indicator of obesity was more strongly associated with the development of T2DM than WC (7). Shao et al. showed that WHtR was better when predicting metabolic syndrome (8). The value of VAI for diagnosing diabetes has been reported in the literature to be higher than BMI and WC (9). Another study among Malaysian vegetarians showed that LAP is more strongly related to metabolic syndrome than other indicators (10). The results of existing studies do not provide conclusive evidence as to which obesity indicator are better for monitoring the new-onset diabetes, and we need to find a more accurate and reliable indicator for the early identification of diabetes. Therefore, we investigated the link between LAP and the new-onset diabetes in a Japanese normoglycemic cohort and compared the ability of LAP to monitor diabetes with other obesity measures (WC, BMI, WHtR, BRI, ABSI, LAP, VAI).

## Materials and methods

### Data source

We obtained the original information from the “Dryad” website (https://datadryad.org/). The site allows people to use the data for secondary research without infringing on the authors’ interests (11).

### Study design and participants

**Figure 1:**
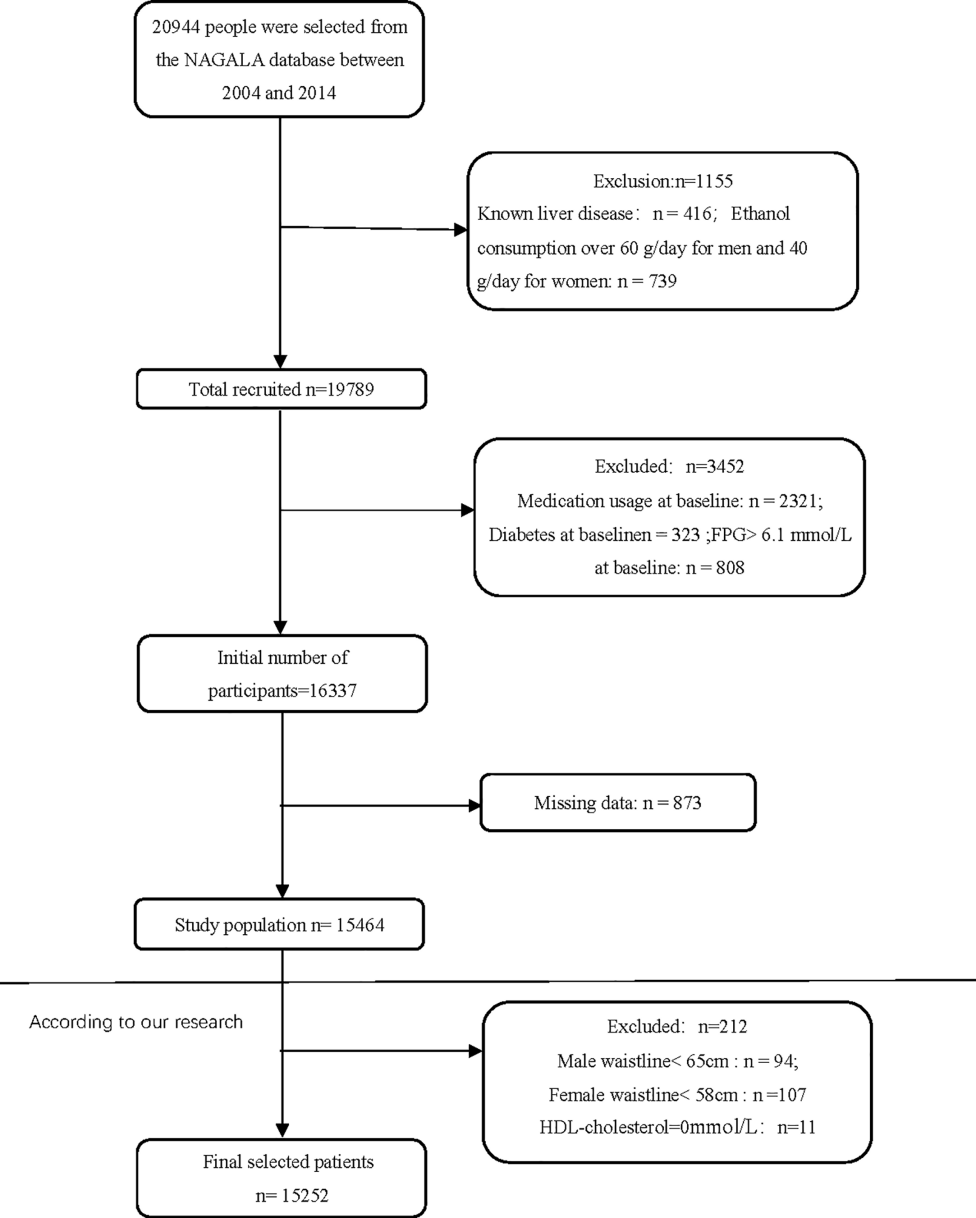
Flowchart of study participants.

The database is from Murakami Memorial Hospital (Gifu, Japan). They performed this search to study chronic disease and its risk factors and improve the level of public health. They collected the medical examination indicators of 20,944 people who participated in the medical examination project from 2004 to 2015. During this period, they also conducted a second examination. Participants with fatty liver, and viral hepatitis, who consumed more than 60g/d of alcohol in men and 40g/d of alcohol in women, at baseline using drugs and T2DM were excluded. Subjects with absent covariates, fasting blood glucose ≥6.1mmol/l, and Hba1c%< 6.5% were excluded (11). The original study included 15464 subjects. Our study excluded subjects with high-density lipoprotein cholesterol (HDL)=0mmol/l, WC<58 cm in women, andWC<65 cm in men (total 212 people). Finally, we had a total of 15252 participants (**Figure 1**).

### Data collection and measurements

All participants completed a survey, underwent a physical examination, and were provided blood for biochemical tests. Demographic characteristics, including age, gender, smoking status, and drinking status, were obtained from the medical records of these individuals. Participants were divided into four groups based on average weekly alcohol intake: no or light drinking, light drinking, moderate drinking, or heavy drinking. People who smoke were classified as smoking, smoking, and never once upon a time. People who exercise regularly during the week are defined as those who exercise regularly. Physical examination measurements included diastolic and systolic blood pressure, height, WC, body weight. Fatty liver was diagnosed by professionals who refer to abdominal ultrasound images. In addition, fasting blood samples were used to measure fasting blood glucose (FBG), hemoglobin A1c (HbA1c), gamma-glutamyl transferase (GGT), aspartate aminotransferase (AST), alanine aminotransferase (ALT), total cholesterol (TC), high-density lipoprotein cholesterol (HDL) and triglyceride (TG).

Waist to height ratio (WHtR)=WC/Height; BRI= $364.2-365.5\times \sqrt{1-{\left(\text{WC}/2\text{π}\right)}^{2}/{\left(0.5\text{height}\right)}^{2}}$ ; ABSI= $\text{WC}÷\left({\text{BMI}}^{2/3}\times {\text{Height}}^{1/2}\right)$ ; VAI (Women)= [WC÷{39.68+(1.88×BMI)}]×[TG(mmol/l)÷1.03]×[1.31÷HDL(mmol/l)] ; VAI (Men)= [WC÷{36.58+(1.89×BMI)}]×[TG(mmol/l)÷0.81]×[1.52÷HDL(mmol/l)] ; LAP (Women)= [(WC (cm))−58]×TG(mmol/l) ; LAP (Men)= [(WC (cm))−65]×TG(mmol/l) (10)

The primary outcome was new-onset diabetes defined by fasting blood glucose ≥ 7mmol/l, HbA1c% ≥ 6.5%, or self-reported.

### Statistical analysis

Continuous variables were presented as means ± standard deviation if normally distributed or as medians (Q1-Q3) if skewed.Categorical variables were expressed as numerical values (percentages). Participants were quartered according to LAP. COX regression analysis showed the results of unadjusted, micro-adjustment, and completely adjusted analysis, and presented as hazard ratio (HR) at a 95% confidence interval (95% CI), and we also calculated the trend of p. Kaplan-Meier curve was used to describe the association between LAP and cumulative diabetes incidence. Using smooth curve fitting to explore the nonlinear association between LAP and diabetes incidence. The threshold effect was analyzed by piecewise linear regression. Receiver operating characteristic (ROC) curve was utilized to assess the capacity of ABSI, BMI, WHtR, BRI, VAI, LAP and WC to monitor diabetes. Then, the area under the curve (AUC) of classical predictors plus obesity indicators was compared with pairs. All tests were two-sided, and a *P*-value<0.05 was considered statistically significant. All the statistical analysis we did was done in R software (version 3.6.3) and EmpowerStats (version 3.0).

## Results

### Basic characteristics of the population

**Table 1** demonstrated the baseline characteristics of participants. This study includes 15252 people, including 8325 men, and 6927 women. During an average follow-up of 6.04 years, there are 87 women and 286 men with diabetes. Quartiles of LAP showed that ALT, fatty liver, TC, age, AST, GGT, TG, HbA1c, SBP, DBP, FPG were higher in the highest LAP quantile group than in the other groups. However, HDL was just the opposite of the above. In addition, among those with higher LAP, our study indicators BMI, WHtR, BRI, VAI, WC and ABSI are also relatively higher. We also observed that the number of four groups of developed diabetes [27 (0.708%), 37 (0.971%), 73 (1.913%), and 236 (6.186%)respectively)] were statistical differences (P<0.001).

**Table 1:** Baseline characteristics of participants (N =15252).

Variables	Q1(<4.890)(n=3812)	Q2 (4.894-9.953) (n=3809)	Q3 (9.958-19.667) (n=3816)	Q4 (19.678-212.582) (n=3815)	P-value
AGE (year)	40.643 ± 8.451	43.130 ± 8.800	45.240 ± 8.737	45.915 ± 8.575	<0.001
alcohol (g/wk)	30.505 ± 63.376	43.639 ± 76.385	51.609 ± 85.608	66.036 ± 96.866	<0.001
BMI (kg/m2)	19.412 ± 1.694	21.215 ± 1.869	22.820 ± 2.183	25.265 ± 2.944	<0.001
WHtR	0.414 ± 0.025	0.450 ± 0.028	0.479 ± 0.032	0.514 ± 0.042	<0.001
BRI	1.907 ± 0.398	2.482 ± 0.480	2.992 ± 0.588	3.661 ± 0.847	<0.001
VAI	0.423 ± 0.221	0.625 ± 0.263	0.965 ± 0.395	2.142 ± 1.369	<0.001
LAP	2.749 ± 1.307	7.225 ± 1.414	14.106 ± 2.762	37.224 ± 19.787	<0.001
WC (cm)	67.261 ± 4.613	73.881 ± 4.931	79.338 ± 5.435	86.291 ± 6.926	<0.001
Follow up duration (days)	2226.957 ± 1371.448	2168.158 ± 1370.988	2189.711 ± 1371.643	2239.715 ± 1400.015	0.087
ALT (IU/L)	14.995 ± 6.715	16.621 ± 8.341	20.191 ± 16.872	28.316 ± 17.922	<0.001
AST (IU/L)	16.816 ± 5.917	17.182 ± 6.166	18.280 ± 11.155	21.331 ± 9.371	<0.001
ABSI	7.322 ± 0.363	7.536 ± 0.369	7.676 ± 0.360	7.752 ± 0.349	<0.001
GGT (IU/L)	14.111 ± 9.916	16.588 ± 12.146	20.727 ± 16.758	30.161 ± 25.547	<0.001
HDL (mmol/L)	1.694 ± 0.389	1.567 ± 0.370	1.404 ± 0.348	1.172 ± 0.286	<0.001
TC (mmol/L)	4.762 ± 0.775	4.980 ± 0.807	5.226 ± 0.810	5.544 ± 0.859	<0.001
SBP (mmHg)	107.136 ± 12.693	111.552 ± 13.229	116.703 ± 13.848	123.106 ± 14.948	<0.001
TG (mmol/L)	0.460 ± 0.216	0.632 ± 0.228	0.891 ± 0.295	1.681 ± 0.820	<0.001
HbA1c (%)	5.110 ± 0.299	5.139 ± 0.305	5.189 ± 0.321	5.251 ± 0.342	<0.001
FPGmmol/L	4.968 ± 0.389	5.091 ± 0.396	5.224 ± 0.377	5.368 ± 0.372	<0.001
DBP (mmHg)	66.434 ± 8.834	69.435 ± 9.478	72.945 ± 9.794	77.839 ± 10.241	<0.001
					<0.001
Women	2524 (66.212%)	2027 (53.216%)	1493 (39.125%)	883 (23.145%)	
Men	1288 (33.788%)	1782 (46.784%)	2323 (60.875%)	2932 (76.855%)	
Fatty liver 0/1					<0.001
No	3776 (99.056%)	3648 (95.773%)	3135 (82.154%)	1956 (51.271%)	
Yes	36 (0.944%)	161 (4.227%)	681 (17.846%)	1859 (48.729%)	
Baseline Habit of exercise					<0.001
No	3094 (81.165%)	3101 (81.412%)	3149 (82.521%)	3240 (84.928%)	
Yes	718 (18.835%)	708 (18.588%)	667 (17.479%)	575 (15.072%)	
Smoking					<0.001
never	2760 (72.403%)	2428 (63.744%)	2041 (53.485%)	1665 (43.644%)	
past	470 (12.329%)	660 (17.327%)	867 (22.720%)	932 (24.430%)	
current	582 (15.268%)	721 (18.929%)	908 (23.795%)	1218 (31.927%)	
Incident DM					<0.001
No	3785 (99.292%)	3772 (99.029%)	3743 (98.087%)	3579 (93.814%)	
Yes	27 (0.708%)	37 (0.971%)	73 (1.913%)	236 (6.186%)	

Values are n (%) or mean ± SD.

BMI, body mass index; WHtR, waist to height ratio; BRI, Body Roundness Index; VAI, visceral Adiposity Index; LAP, lipid accumulation product; WC, waist circumference; ABSI, A Body Shape Index; SBP, systolic blood pressure; DBP, diastolic blood pressure; FPG, fasting plasma glucose; HbA1c, hemoglobinA1c; TC, total cholesterol; TG, triglyceride; HDL-C, high-density lipoprotein cholesterol; AST, aspartate aminotransferase; ALT, alanine aminotransferase; GGT, gamma-glutamyl transferase.

### Kaplan–Meier curves of time to incident diabetes during follow-up

**Figure 2:**
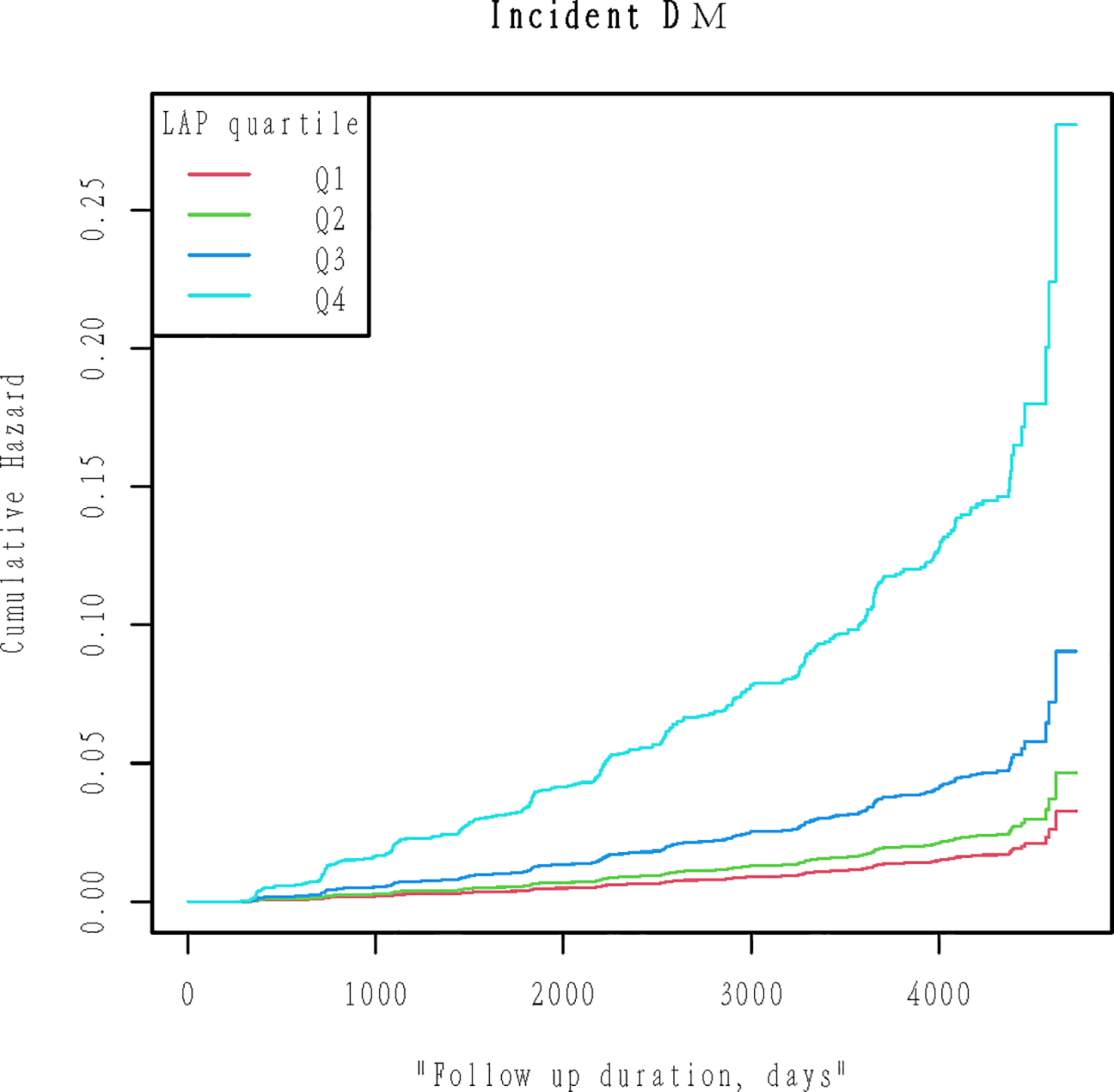
Kaplan-Meier plots of cumulative diabetes incidence for LAP quartiles groups during follow-up. Likelihood ratio test=257.4, p< 0.001.

By the end of the follow-up, a total of 373 (2.446%) people had diabetes. We quadrupled the LAP and compared the cumulative hazard of the four groups. **Figure 2** Kaplan-Meier curve was used to describe the association between LAP and cumulative hazard. As shown in **Figure 2**, cumulative hazard increases with the increase of LAP, and the group with the highest LAP is significantly different from the other three groups (Likelihood ratio test=257.4, P<0.001).

### Multivariable COX proportional hazards regression analysis of the association between baseline LAP and incident diabetes

Multivariate regression analyses was employed to evaluate the impact of baseline LAP on new-onset diabetes (**Table 2**), including crude models and adjusted models. It can be seen that it was positively associated with the onset of diabetes when LAP was treated as a continuous variable. We further treated LAP as a categorical variable, and as the quantile of LAP increased, the prevalence of diabetes increased in the crude model (P for trend<0.001). The highest group than the lowest incidence of diabetes increased by 761.4% (HR:8.614 95% CI: 5.785 to 12.828, P<0.001). In model 2, we adjusted for gender and age, the HR for Q2-Q4 were HR=1.229, 95% CI: (0.746, 2.024), HR=2.142 95% CI: (1.366, 3.359), HR=6.161 95% CI: (4.064, 9.341) respectively (P for trend<0.001). Model 2 was further adjusted for the habit of exercise, ethanol consumption, smoking status, SBP, and DBP based on model 1. The strong association between LAP and diabetes was still observed (Q2 HR=1.150, 95% CI: 0.697 to 1.895, Q3 HR=1.870, 95% CI: 1.188 to 2.942, Q4 HR=4.813, 95% CI: 3.146 to 7.365). Based on model 2, ALT, AST, HbA1c, FPG, and GGT were further modulated. In model 3, as compared to Q1, the hazard ratio of diabetes in the Q2, Q3, and Q4 were HR: 0.855, 95% CI: (0.514, 1.424), HR: 1.172, 95% CI: (0.744, 1.846), HR: 1.768, 95% CI: (1.139, 2.746) respectively (P for trend was<0.0001). We can also find that in Q4 P<0.05 and Q2 and Q3 groups, P>0.05, is not significant. It is suggested that nonlinear relationship exists between LAP and diabetes prevalence. The **Supplementary Table 1** shows that LAP is more strongly associated with new-onset diabetes in women, those with fatty liver, and current smokers.

**Table 2:** Multivariable COX proportional hazards regression analysis of the association between baseline LAP and incident diabetes.

Incident diabetes	Crude Model	Model 1	Model 2	Model 3
	HR (95% CI) *P*-value	HR (95% CI) *P*-value	HR (95% CI) *P*-value	HR (95% CI) *P*-value
LAP	1.028 (1.026, 1.031) 0.001	1.026 (1.023, 1.029)<0.001	1.023 (1.020, 1.027)<0.001	1.015 (1.011, 1.019)<0.001
LAP quartile				
Q1	Ref	Ref	Ref	Ref
Q2	1.429 (0.870, 2.346) 0.159	1.229 (0.746, 2.024) 0.418	1.150 (0.697, 1.895) 0.585	0.855 (0.514, 1.424) 0.548
Q3	2.775 (1.785, 4.316)<0.001	2.142 (1.366, 3.359)<0.001	1.870 (1.188, 2.942) 0.007	1.172 (0.744, 1.846) 0.495
Q4	8.614 (5.785, 12.828)<0.001	6.161 (4.064, 9.341)<0.001	4.813 (3.146, 7.365)<0.001	1.768 (1.139, 2.746) 0.011
P for trend	<0.001	<0.001	<0.001	<0.001

Crude model: we did not adjust other covariates.

Model 1: we adjusted age, sex.

Model 2: we adjusted age, sex, Habit of exercise, ethanol consumption, smoking status, SBP, DBP.

Model 3: we adjusted age, sex, Habit of exercise, ethanol consumption, smoking status, SBP, DBP, ALT, AST, HbA1c,%, FPG, GGT.

HR, Hazard ratio; CI, confidence interval; Ref, Reference; LAP, lipid accumulation product; SBP, systolic blood pressure; DBP, diastolic blood pressure; FPG, fasting plasma glucose; HbA1c, hemoglobinA1c; AST, aspartate aminotransferase; ALT, alanine aminotransferase; GGT, gamma-glutamyl transferase.

### Analysis of threshold effects of LAP and new onset diabetes mellitus

Since LAP is a continuous variable, a smooth curve fitting was utilized to explore the connection between LAP and diabetes. After adjusting the variables including age, sex, Habit of exercise, ethanol consumption, smoking status, SBP, DBP, ALT, AST, HbA1c%, FPG, and GGT, The link between baseline LAP and new-onset diabetes is non-linear, logarithmic likelihood ratio P=0.012 (**Table 3**). When LAP was less than 46.402, HR: 1.025, 95% CI: 1.016-1.034, P<0.001. For LAP of 46.402 or more HR: 1.008, 95% CI: 1.001-1.015, P =0.0128.

**Table 3:** Threshold Effect Analysis of WC and diabetes using Piece-wise Linear Regression.

Incident diabetes	HR, 95% CI	P-value
**Fitting model by standard linear regression**	**1.015 (1.011, 1.019)**	**<0.0001**
**Cutoff point (K)**	**46.402**	
**<46.38**	**1.025 (1.016, 1.034)**	**<0.0001**
**≥46.38**	**1.008 (1.001, 1.015)**	**0.0128**
**Logarithmic likelihood ratio**	**0.012**	

Adjusted for age, sex, Habit of exercise, ethanol consumption, smoking status, SBP, DBP, ALT, AST, HbA1c, %, FPG; GGT.

### Receiver−operating characteristics curve analysis

**Figure 3:**
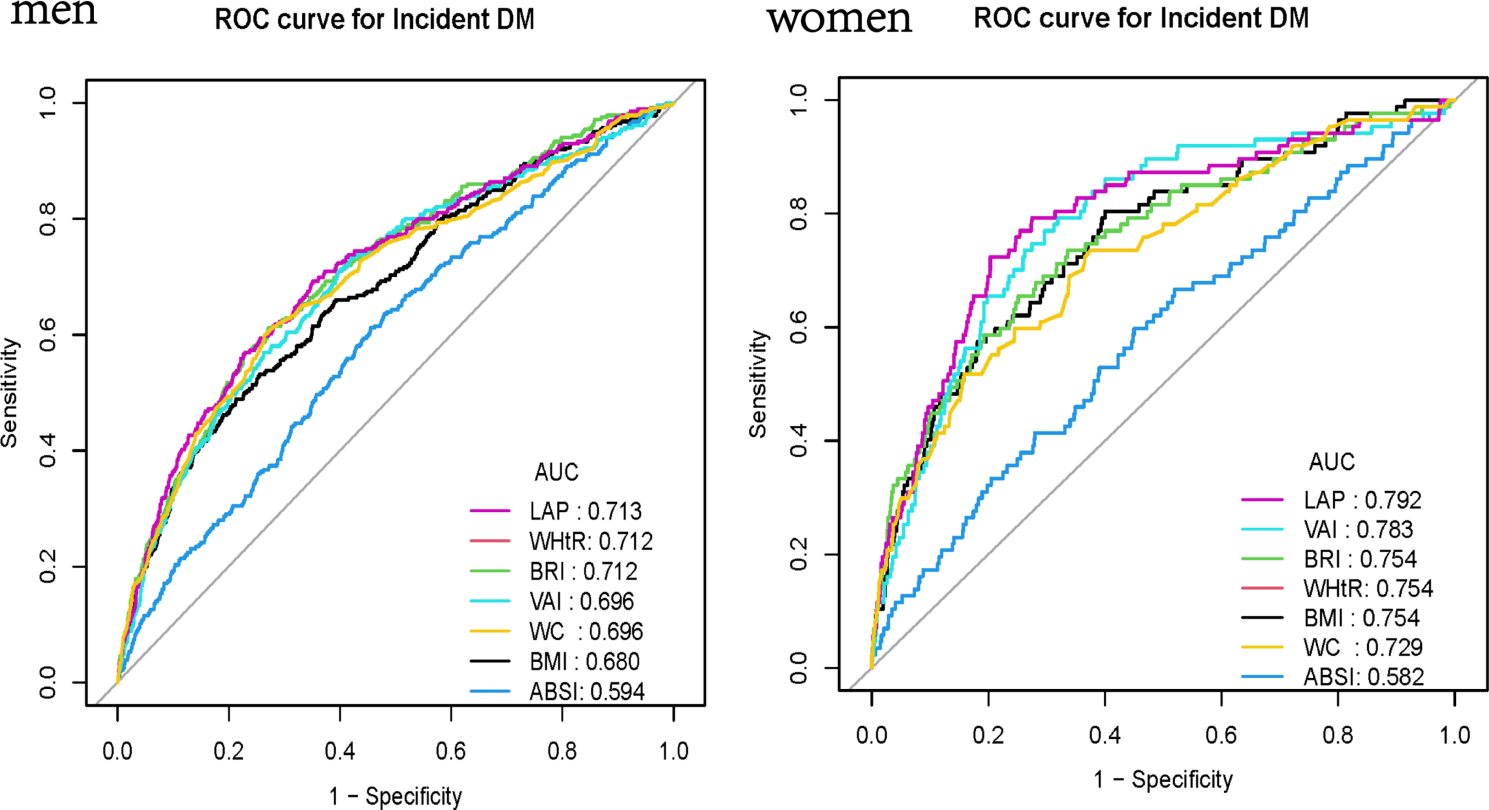
ROC curve analysis of BMI, WHtR, BRI, VAI, LAP, WC and ABSI in men and women.

**Figure 4:**
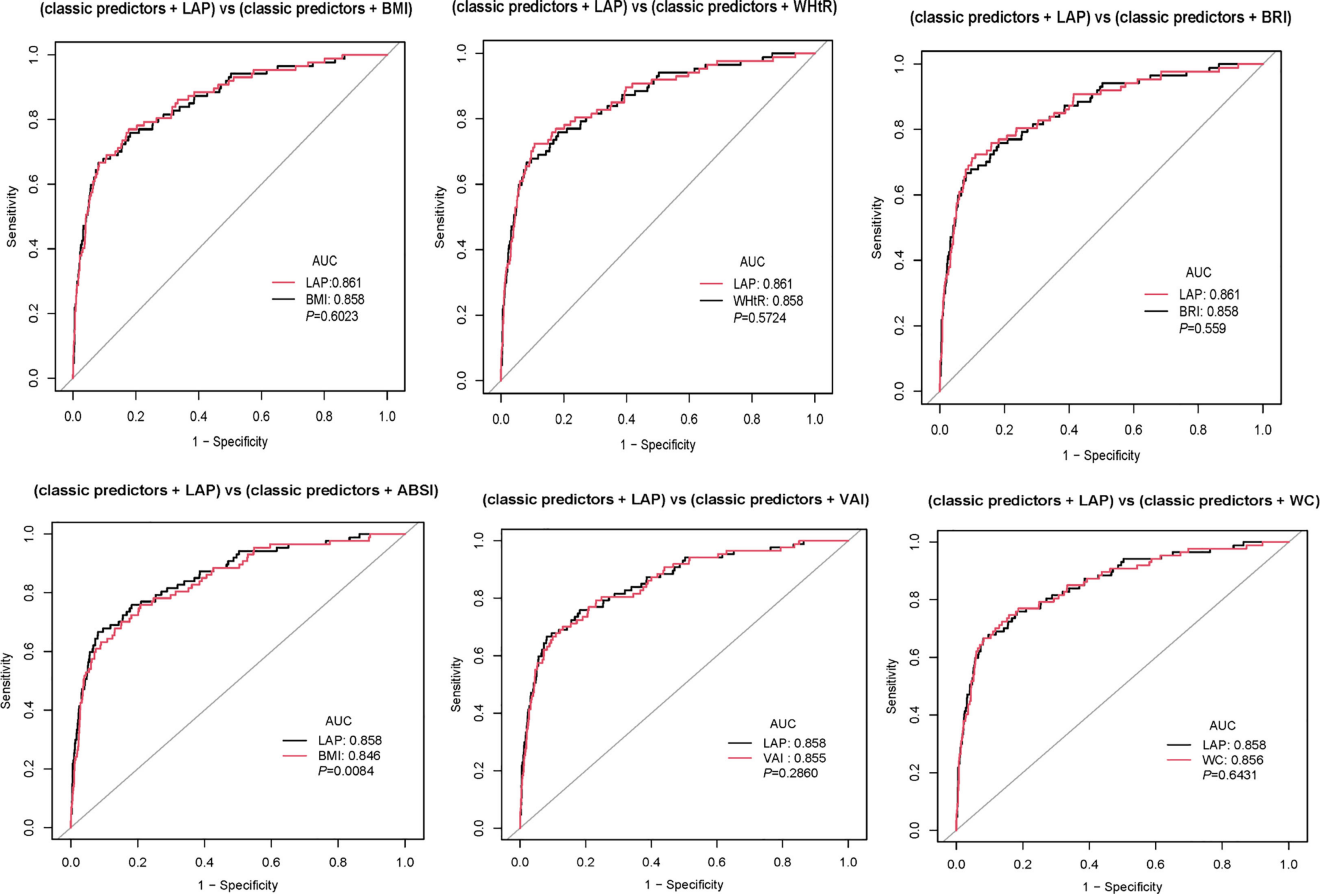
Pairwise comparison of AUC between LAP and other parameters in women.

**Figure 5:**
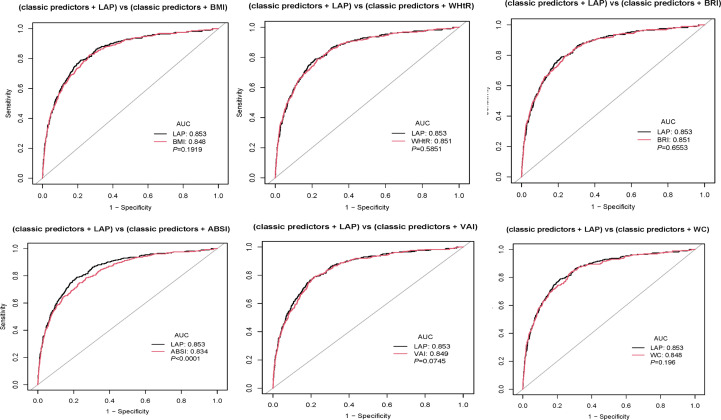
Pairwise comparison of AUC between LAP and other parameters in men.

ROC curves were adopted to measure the forecast value. From **Figure 3**, we can see that LAP has the largest AUC in females. In males, however, the curve that represents the LAP does not show a significant advantage. In females, the AUC of BMI, WHtR, BRI, ABSI, VAI, LAP, and WC were 0.6802(0.6469-0.7136), 0.7118(0.6801-0.7435), 0.7118(0.6801-0.7435), 0.5943(0.5608-0.6277), 0.6962(0.6634-0.7291), 0.713(0.6806-0.7454), 0.6961(0.6624-0.7298), respectively. In males, the AUC of BMI, WHtR, BRI, ABSI, VAI, LAP, and WC were 0.7539(0.6999-0.8080), 0.7543(0.698-0.8106), 0.7543(0.698-0.8106), 0.5821(0.5196-0.6447), 0.783(0.7331-0.8328), 0.7922(0.7396-0.8447), 0.7287(0.6714-0.786) respectively (**Table 4**). Their P values are significant (all P<0.001). Furthermore, classical predictors FBG, HbA1c, SBP, age, exercise, and obesity indicators already mentioned were added to the model for comparison. (**Figure 4**), We can see that in both men and women, LAP with classical predictors predicts the incidence of diabetes better than ABSI with classical predictors, but not significantly different from the other parameters (**Figure 5**).

**Table 4:** ROC curves of BMI, WHtR, BRI, VAI, LAP, WC, ABSI for predicting diabetes in men and women.

		AUC(95%CI)	Specificity(%)	Sensitivity(%)	*P* value
Men	BMI	0.7539 (0.6999-0.8080)	0.6	0.8046	<0.001
	WHtR	0.7543 (0.698-0.8106)	0.7488	0.6552	<0.001
	BRI	0.7543 (0.698-0.8106)	0.7488	0.6552	<0.001
	ABSI	0.5821 (0.5196-0.6447)	0.5501	0.5977	<0.001
	VAI	0.783 (0.7331-0.8328)	0.6811	0.7931	<0.001
	LAP	0.7922 (0.7396-0.8447)	0.7966	0.7241	<0.001
	WC	0.7287 (0.6714-0.786)	0.6246	0.7356	<0.001
Women	BMI	0.6802 (0.6469-0.7136)	0.7869	0.4895	<0.001
	WHtR	0.7118 (0.6801-0.7435)	0.7289	0.6119	<0.001
	BRI	0.7118 (0.6801-0.7435)	0.7289	0.6119	<0.001
	ABSI	0.5943 (0.5608-0.6277)	0.5452	0.6119	<0.001
	VAI	0.6962 (0.6634-0.7291)	0.5802	0.7343	<0.001
	LAP	0.713 (0.6806-0.7454)	0.6492	0.6923	<0.001
	WC	0.6961 (0.6624-0.7298)	0.7344	0.6014	<0.001

ROC, receiver-operating characteristics; AUC: area under the curve; CI, confidence interval; BMI, body mass index; WHtR, waist to height ratio; BRI, Body Roundness Index; VAI, visceral Adiposity Index; LAP, lipid accumulation product; WC, waist circumference; ABSI, A Body Shape Index.

## Discussion

This retrospective study based on the Japanese population revealed that the cumulative incidence of diabetes increased with the elevation of LAP (HR=1.768, 95% CI: 1.139 to 2.746, *P*=0.011). Baseline LAP has the largest AUC compared to other indicators, showing a strong diagnostic value for diabetes, especially in women.

Obesity is closely related to diabetes and is also one of the common indicators used to predict diabetes in clinical practice. As people’s living standards have improved in recent years, obesity has also increased rapidly, which is one of the reasons for the rapid rise in diabetes (12). Many obesity indicators have been developed for early diagnosis of diabetes. BMI represents general obesity, but it did not reflect the body fat distribution. For example, the number of people with type 2 diabetes(T2DM) in Japan has been climbing, but the annual change in average BMI has decreased (11). WC does not clearly distinguish between subcutaneous and visceral fat in the abdominal cavity (13). Moreover, we all know that people of different heights with the same WC have different body fat percentages. Therefore, WHtR, BRI, and ABSI, which combine WC and height, seem more appropriate to predict diabetes. Multiple previous studies have shown that fat from abdominal organs is more strongly associated with diabetes, hypertension, and cardiovascular disease than subcutaneous tissue obesity (14–16). Obesity-induced inflammatory response is one of the key triggers of T2DM (17). In overweight individuals, chronic overnutrition and chronic inflammation of adipose tissue can contribute to insulin resistance (IR) and impaired glucose metabolism (18). IR is a critical step in the process of incident diabetes (19), and obesity-induced chronic inflammation and mitochondrial dysfunction both exacerbate insulin resistance (20–22). Additionally, long-term exposure to fatty acids in beta cells reduces glucose-induced insulin secretion, impairs insulin gene expression, and increases cell death (23, 24). The onset of diabetes is very insidious and complications may have already occurred by the time it is detected, so its early detection and prevention are particularly important. Some studies have used magnetic resonance imaging (MRI) to assess abdominal obesity and visceral fat obesity. It is undeniable that this can provide a more precise understanding of body fat division (25). However, in reality, few people have regular MRIs to detect body fat distribution. Therefore new obesity indicators LAP and VAI have been developed. They are indicators that are a combination of body measurements and blood test indicators. Multiple studies have shown a robust relationship between LAP, VAI and metabolic syndrome, and diabetes (26, 27). Weight loss can reverse to some extent the underlying metabolic abnormalities caused by type 2 diabetes, resulting in better glycemic control (28). Moreover, LAP is also relevant to diabetic complications such as diabetic nephropathy, diabetic retinopathy, and cardiovascular risk (29–31). Considering the high prevalence rate, serious complications, and serious social and economic burden caused by type 2 diabetes, it is very important to find a low-cost, easy-to-operate, and easy-to-calculate indicator to identify diabetes as early as possible and conduct lifestyle or drug intervention if necessary.

In a 12-year follow-up study that included 8,281 Korean participants aged 40-69 years, a very strong correlation was noted between LAP and diabetes [HR=2.14, 95%CI:1.56-2.94]. This research demonstrated the stronger predictive power of LAP than BMI and WC (32), consistent with our findings. However, this study has two weaknesses compared with our study. On the one hand, the study only selected non-obese people with WC<85 cm in famales,<90 cm in males, BMI<25 kg/m2, and not taking lipid-lowering medications, whereas our study population included this obese population. On the other hand, the study was conducted in the middle-aged and elderly population, whereas our study included people aged 18-79 years. A survey of the Chinese population suggested that VAI outperformed by LAP, WC and BMI as a predictor of metabolic syndrome (9). Our results show that LAP has the largest AUC (0.7922, 95% CI: 0.7396-0.8447 in men, 0.713, 95% CI: 0.6806-0.7454 in women) and higher predictive value compared to other metrics. The results of the two studies differed, probably because of the different populations selected. This study in a Chinese population selected 537 people with chronic kidney disease, while we selected 15,252 people with blood glucose less than 6mmol/l. Our study population was larger and the results were more stable. An investigation among Brazilians showed that LAP monitors metabolic syndrome better than BMI, WC, and WHtR (33). In line with our results, we recruited more participants and we also measured glycated hemoglobin, making the results more reliable. Our results indicated that LAP had the largest AUC and high specificity and sensitivity, suggesting that LAP can be used for the surveillance of people vulnerable to diabetes. The strong correlation of LAP can be explained by the close relationship between its constituents and insulin resistance. Both WC and TG are independent risk factors for diabetes and are pivotal in the pathogenesis of DM (34, 35). In combination with our research, we believe that LAP is a more comprehensive and credible indicator for monitoring diabetes among obesity indicators[HR=1.768, 95% CI: (1.139-2.746), *P*=0.011]. The strength of this study lies in its novelty, revealing a strong association between LAP and new-onset diabetes. The ROC curve showed that LAP was better than other obesity indicators. Finally, we add classical predictors into the above models and compare them in pairs.

Of course, there were some limitations in this study. First, the study was conducted in a Japanese population and may not be suitable for generalization to other ethnic groups due to differences in race, age, and lifestyle habits. Second, the main outcome indicators are defined according to fasting blood glucose≥ 7mmol/l, HbA1c% ≥ 6.5%, or self-reported, without performing an oral glucose tolerance test, which may underestimate the prevalence of diabetes to some extent. Finally, because this was a secondary analysis study, we were unable to adjust for variables that were not measured in the original data, which could have potentially affected the results.

## Conclusions

In conclusion, we proved that LAP is the independent risk factor of diabetes in the Japanese population, especially among women, those with fatty liver, and current smokers. And found that the AUC was greatest when LAP alone predicted diabetes in both women and men. But when the classical predictors are included, the LAP is no better than any other indicator, except the ABSI. Therefore, LAP may be a worthwhile indicator to consider when using a single indicator of obesity to screen for susceptibility to diabetes.

## Data availability statement

The original contributions presented in the study are included in the article/**Supplementary Material**. Further inquiries can be directed to the corresponding author.

## Ethics statement

The studies involving human participants were reviewed and approved by the Ethics Committee of Murakami Memorial Hospital. The patients/participants provided their written informed consent to participate in this study.

## Author contributions

TL and WL and CL designed the study. TL and WL collected the data. TL, CL and WL analyzed the data. XZ, TY, WL, GG, HF, and BS interpreted the result. TL wrote the first draft of the manuscript. WL contributed to the refinement of the manuscript. TL and WL contributed equally to this work and share the first authorship. All authors contributed to the article and approved the submitted version.
