# Supplementary material for: Relationship between lipid accumulation product and new-onset diabetes in the Japanese population: a retrospective cohort study
Source: Front Endocrinol (Lausanne). 2023 May 17;14:1181941. doi: 10.3389/fendo.2023.1181941 (PMC10230034; doi:10.3389/fendo.2023.1181941)
Supplement: Supplementary file 1 [file DataSheet_1.pdf]

Supplementary Material

Supplementary table 1.

Supplementary table 1 efect size of LAP on DM in prespecifed and exploratory subgroups

Characteristic	No. of participants	HR (95% CI)	*P*-value	*P* for interaction
**SEX**				**0.0125**
**Women**	**6927**	**1.032(1.023,1.045)**	**<0.0001**	
**Men**	**8325**	**1.018(1.014,1.026**	**<0.0001**	
**Fatty liver**				**0.486**
**No**	**12515**	**1.007 (0.997, 1.017)**	**0.1813**	
**Yes**	**2737**	**1.012 (1.007, 1.016)**	**<0.0001**	
**Age（years）**				**0.25**
**<39**	**4755**	**1.016 (1.007, 1.025)**	**0.0003**	
**39-47**	**5059**	**1.019 (1.012, 1.026)**	**<0.0001**	
**≥47**	**5438**	**1.011 (1.005, 1.017)**	**0.0002**	
**DBP (mmHg)**				**0.094**
**<90**	**14491**	**1.017 (1.013, 1.021)**	**<0.0001**	

**>=90**	**761**	**1.007 (0.997, 1.016)**	**0.1612**	
**SBP (mmHg)**				**0.028**
**<140**	**14470**	**1.017 (1.013, 1.021)**	**<0.0001**	
**>=140**	**782**	**1.006 (0.996, 1.015)**	**0.2447**	
**Smoking status**				**0.7504**
**never**	**8894**	**1.015 (1.008, 1.021)**	**<0.0001**	
**past**	**2929**	**1.013 (1.003, 1.023)**	**0.0092**	
**current**	**3429**	**1.016 (1.009, 1.022)**	**<0.0001**	
**Habit of Exercise**				**0.474**
**No**	**12584**	**1.014 (1.010, 1.018)**	**<0.0001**	
**Yes**	**2668**	**1.023 (1.013, 1.034)**	**<0.0001**	

Abbreviations: SBP, systolic blood pressure; DBP, diastolic blood pressure

Notes: adjusted for age, sex, Habit of exercise, ethanol consumption, smoking status, SBP, DBP, ALT, AST, HbA1c,%, FPG , GGT

Supplementary Figure 1 A bar graph showing the changes and dependence of LPA and diabetes markers.

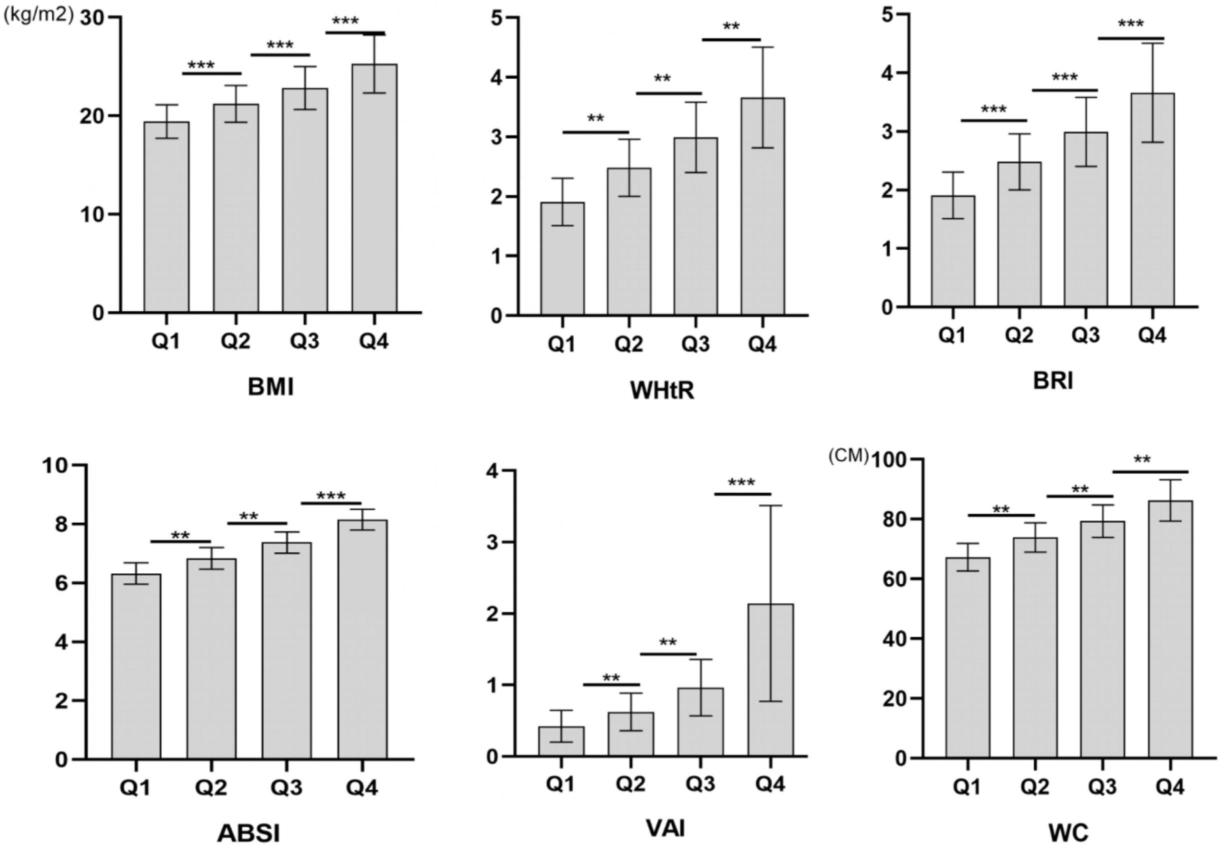
(kg/m2)
30
20
10
0
***
***
***
Q1
Q2
Q3
Q4
BMI
5
4
3
2
1
0
**
**
**
Q1
Q2
Q3
Q4
WHtR
5
4
3
2
1
0
***
***
***
Q1
Q2
Q3
Q4
BRI
10
8
6
4
2
0
**
**
***
Q1
Q2
Q3
Q4
ABSI
4
3
2
1
0
**
**
***
Q1
Q2
Q3
Q4
VAI
(CM)
100
80
60
40
20
0
**
**
**
Q1
Q2
Q3
Q4
WC
